# Recovery Performance of Ge-Doped Vertical GaN Schottky Barrier Diodes

**DOI:** 10.1186/s11671-019-2872-7

**Published:** 2019-01-31

**Authors:** Hong Gu, Feifei Tian, Chunyu Zhang, Ke Xu, Jiale Wang, Yong Chen, Xuanhua Deng, Xinke Liu

**Affiliations:** 1 College of Materials Science and Engineering, Guangdong Research Center for Interfacial Engineering of Functional Materials, Shenzhen Key Laboratory of Special Functional Materials, Chinese Engineering and Research Institute of Microelectronics, Shenzhen University, Shenzhen, 518060 People’s Republic of China; 2 Key Laboratory of Optoelectronic Devices and Systems of Ministry of Education and Guangdong Province, College of Optoelectronic Engineering, Shenzhen University, Shenzhen, 518060 People’s Republic of China; 3 Suzhou Institute of Nano-Tech and Nano-Bionics, CAS, Suzhou, 215123 People’s Republic of China

**Keywords:** Vertical SBDs, Ge-doped GaN substrates, Reverse recovery time, HVPE

## Abstract

Vertical GaN Schottky barrier diodes (SBDs) were fabricated on Ge-doped free-standing GaN substrates. The crystal quality of the SBDs was characterized by cathode luminescence measurement, and the dislocation density was determined to be ~ 1.3 × 10^6^ cm^− 2^. With the electrical performance measurements conducted, the SBDs show a low turn-on voltage *V*_on_ (0.70~0.78 V) and high current *I*_on_/*I*_off_ ratio (9.9 × 10^7^~1.3 × 10^10^). The reverse recovery characteristics were investigated. The reverse recovery time was obtained to be 15.8, 16.2, 18.1, 21.22, and 24.5 ns for the 100-, 200-, 300-, 400-, and 500-μm-diameter SBDs, respectively. Meanwhile, the reverse recovery time and reverse recovery charge both show a significant positive correlation with the electrode area.

## Introduction

Recently, a wide band gap semiconductor—such as GaN—with the inherent advantages, has attracted tremendous research attention for the next-generation electronics devices, particularly in the field of high frequency, high power, and high performance [1–6]. Meanwhile, thanks to the developments of hydride vapor phase epitaxy (HVPE), low dislocation density (≤ 10^6^ cm^− 2^) GaN substrates are now commercially available [7–10]. Compared with lateral devices, vertical-type devices fabricated with these substrates are considered to be a more advanced structure which is conducive to achieving a larger current, less leakage path, and better reliability for the system [11, 12]. Among them, GaN-based Schottky barrier diode (SBD) is a vital component in the switching devices. Differed from a bipolar diode, the SBD with its unipolar nature greatly reduces the minority carrier storage effect and correspondingly offers a high switching speed with low reverse recovery loss. However, few groups have conducted a systematic study of the reverse recovery characteristics for vertical GaN SBDs [13–17], of which studies focused more on the comparison of the switching time in different structures devices. Thus, there is still an urgent need of a deep investigation into the mechanism of recovery performance for GaN SBDs, especially for the vertical ones.

Meanwhile, since the ohmic contact technique has been continuously explored to improve device performance in many published papers [18], heavily doped n-type GaN is a key link for fabricating nitride devices. Lately, Ge is proposed as an alternative to Si dopant in GaN, because both of them share a similar characteristic of shallow level impurity (the activation energy is reported to be 20 and 17 meV for Ge and Si, respectively) and the lattice distortion caused by Ge atoms substituting into Ga sites would be smaller owing to their closer ionic radii [19, 20]. The Ge doping is believed to form a smoother surface with fewer defects [21, 22]. Moreover, with the lower lattice distortion and film stress, this doping also shows a promise in high-temperature electronic devices that put more emphasis on the thermal stability. Although the Ge-doped GaN has been studied theoretically, it is essential to investigate the real impact on the relevant device. In this paper, the novel vertical GaN SBDs fabricated on Ge-doped free-standing (FS) GaN substrate are proposed. The vertical GaN SBDs exhibit a superior crystal quality and electronic property. Meanwhile, the recovery performance of vertical SBDs is systematically investigated. The reverse recovery time and reverse recovery charge finally show a significant positive correlation with the electrode area.

## Methods and Experiments

**Fig. 1:**
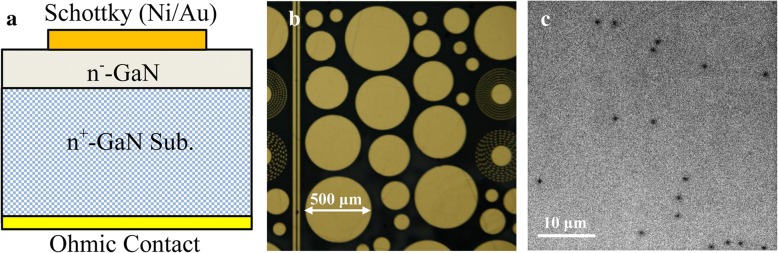
**a** Schematic cross section of the fabricated vertical SBD. **b** Optical microscopy image of the different electrodes. **c** Panchromatic CL image of the epitaxial layer

The schematic of device structures for the fabricated SBDs is displayed in Fig. 1a, which mainly consists of a 390-μm FS n^+^-GaN substrate and a 9-μm n^−^-GaN drift layer. In this work, the (0001)-oriented GaN substrate layer with a Ge concentration of 1 × 10^18^ cm^− 3^ and a dislocation density of 1 × 10^6^ cm^− 2^ was grown by HVPE. And the undoped epitaxial layer on this substrate was grown by metalorganic chemical vapor deposition, with a growth rate of ~ 2 μm/h. For SBD fabrication, Ti/Al/Ni/Au ohmic contacts were formed on the back surface of the GaN substrate. Ni/Au Schottky electrodes were formed on the front surface of the epitaxial layer with five different diameters (100, 200, 300, 400, and 500 μm), as shown in Fig. 1b. More information about the fabrication process can be found in our previous report [23, 24].

The cathodoluminescence (CL) images were obtained using a Quanta 400 FEG scanning electron microscope (SEM) with a 10-kV accelerating voltage to study the spatial distribution of dislocation density for the epitaxial layer. Capacitance-voltage (*C-V*) and current-voltage (*I-V*) measurements were performed using a Keithley 4200 semiconductor parameter analyzer to evaluate electronic properties of the SBDs. And temperature-dependent measurements were conducted in the range of 300 to 500 K with a customized experimental setup.

## Results and Discussion

The CL result of the epitaxial layer is presented in Fig. 1c. As the dislocation is believed to be a nonradiative recombination center, it appears on the CL image in the form of a dark spot. Since no noticeable spatial distribution difference is observed, the average value of dislocation density was calculated to be ~ 1.3 × 10^6^ cm^− 2^, with the CL measurements performed at several different regions. This result indicates a high-quality epitaxial layer was obtained for vertical SBDs.

**Fig. 2:**
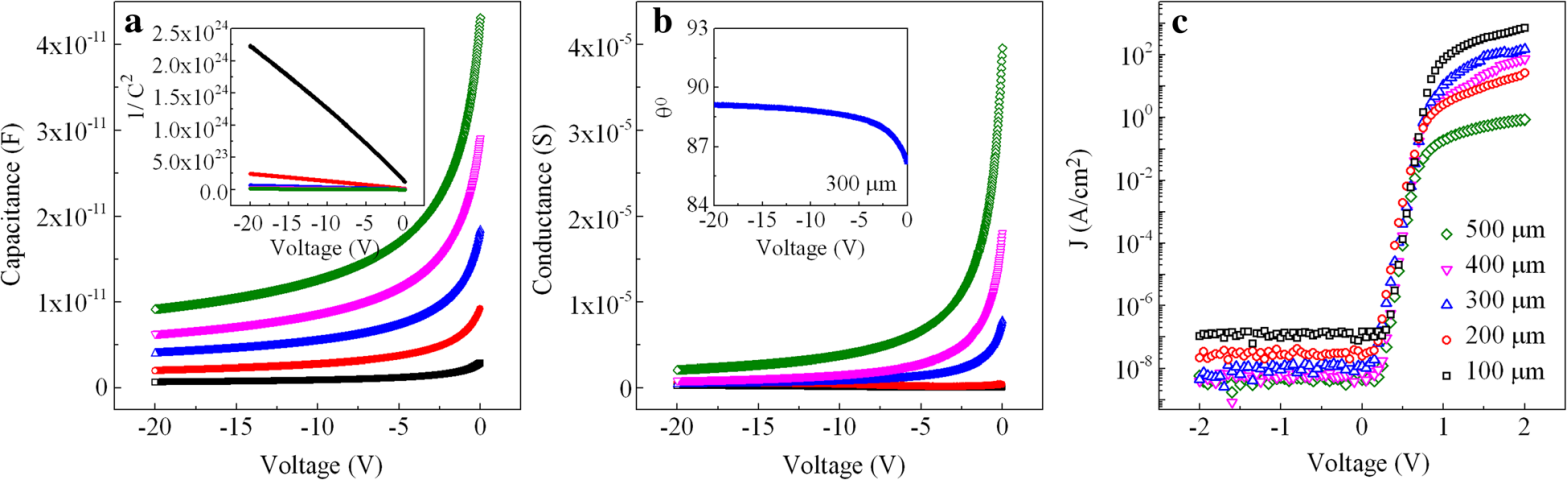
**a** Room-temperature *C-V* curve of vertical SBDs for each different electrode at a frequency of 1 MHz. The inset is a plot of (*1/C*^2^) versus voltage *V*. **b** *G-V* curve of vertical SBDs for each different electrode. The inset is a plot of phase angle *θ* versus voltage *V* for 300-μm-diameter SBDs. **c** *J-V* curve of vertical SBDs for each different electrode

As the vertical SBDs were characterized in a parallel mode, the *C-V* and *G-V* curves were obtained with 1 MHz frequency. The results of the SBDs are shown in Fig. 2a and b, respectively, where (*1/C*^2^) versus applied voltage *V* is plotted in the inset. Here, carrier concentration *N*_*d*_ could be evaluated with the expression: ${N}_d=\frac{-2}{A^2 q\varepsilon \left[d\left(1/{C}^2\right)/ dV\right]}$, where *A* is the area of Schottky electrodes, *q* is the electron charge (1.602 × 10^− 19^ C), and *ε* is the dielectric constant of GaN (8.854 × 10^− 11^ F/m). Hence, the *N*_*d*_ of the epitaxial layer was determined to be ~ 6.2 × 10^15^ cm^− 3^. And the phase angle *θ* also could be calculated by the following equation: $\theta ={\tan}^{-1}\left(\frac{2\pi fC}{G}\right)$, where *f* is the applied frequency, *C* is the capacitance, and *G* is the measured conductance (gate leakage). Since the results for different diameters are similar, the calculated angle *θ* versus applied voltage *V* of the 300-μm-diameter SBDs is shown in the inset of Fig. 2b as an example. Note that the *θ* is very close to 90°, it confirms that an excellent Schottky gate with a low leakage path is achieved in this study. The *J-V* characteristics are also presented in Fig. 2c. It is clearly seen that the *I*_on_/*I*_off_ ratios are 3.8 × 10^9^, 5.9 × 10^8^, 1.3 × 10^10^, 6.5 × 10^8^, and 9.9 × 10^7^ for the 100-, 200-, 300-, 400-, and 500-μm-diameter SBDs, respectively, of which the *I*_on_ and *I*_off_ are defined as the current at the gate voltage of 1.6 and − 2 V, respectively. After linear fitting, the turn-on voltage *V*_on_ of vertical SBDs is determined to be 0.70, 0.76, 0.72, 0.70, and 0.78 V, respectively, with the electrode diameters increasing from 100 to 500 μm. These results indicate a good electronic property was obtained for the vertical SBDs.

**Fig. 3:**
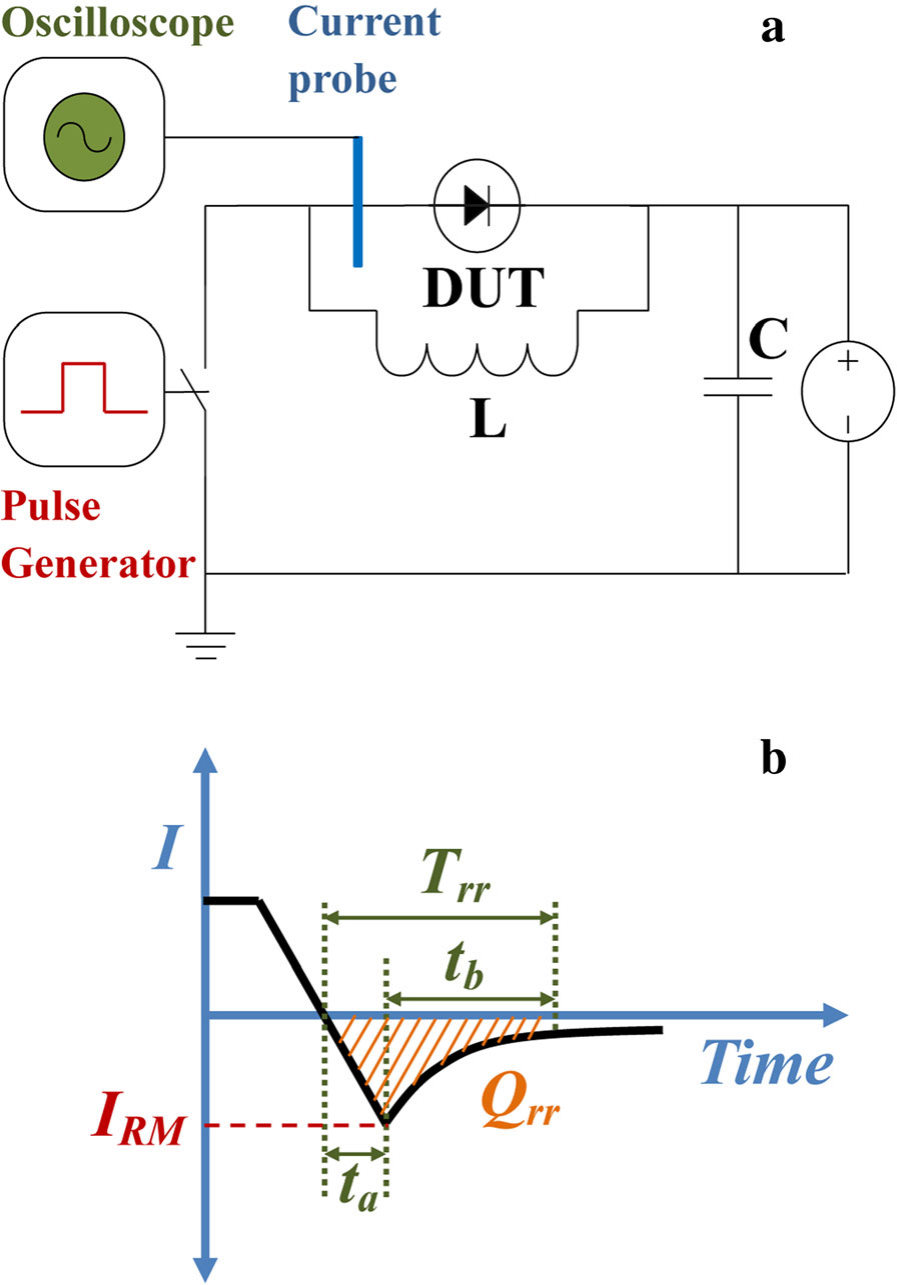
**a** The test circuit used to measure the reverse recovery characteristics of vertical SBDs. **b** Schematic waveform of reverse recovery characteristics of vertical SBDs

A typical test circuit was used to measure the reverse recovery characteristics of the vertical SBDs, as shown in Fig. 3a. The periodic square wave voltage signals (from + 20 to − 20 V) were applied sequentially to a device under test (DUT), where a parasitically inductor would store the magnetic energy and affect the current. When the voltage signal changed, an oscillation current may take place during the period. A high-speed current probe with a Tektronix MDO 4104-3 oscilloscope was disposed for detecting the transient current variation in the vertical SBDs. As the schematic waveform of reverse recovery current is shown in Fig. 3b, in this study, *t*_*a*_ is defined as the storage time while *t*_*b*_ is defined as the reverse current delay time. And the reverse recovery time *T*_*rr*_ is defined as the time when the reverse current recovers to 10% of the maximum reverse recovery current *I*_*RM*_, which is the sum of *t*_*a*_ and *t*_*b*_. And the reverse recovery charge *Q*_*rr*_ is obtained by integrating the reverse current until *T*_*rr*_ which corresponds to the accumulated charge in a diode.

**Fig. 4:**
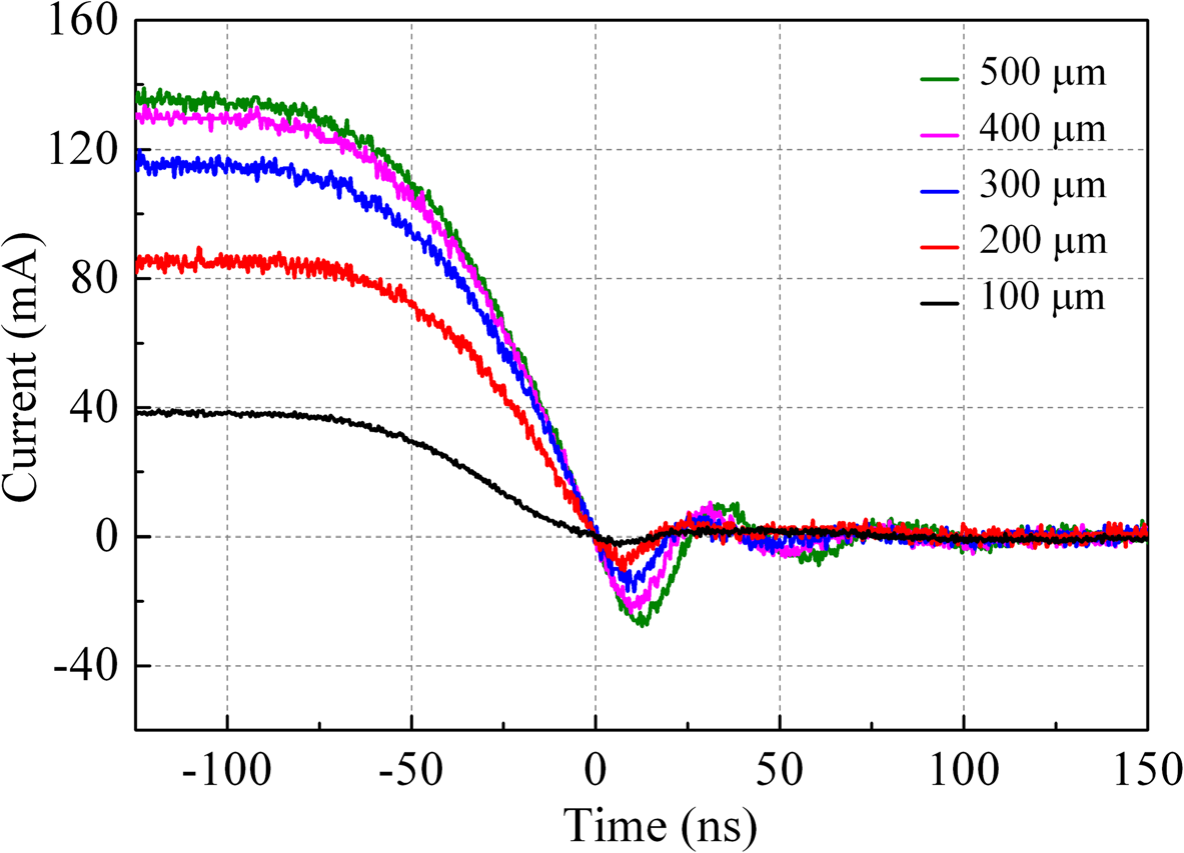
Reverse recovery current of vertical SBDs for each different electrode

Figure 4 shows the reverse recovery curve of vertical SBDs for each electrode diameter when the applied voltage switched from + 20 to − 20 V. Here, for the 100-, 200-, 300-, 400-, and 500-μm-diameter SBDs, the *T*_*rr*_ values were obtained to be 15.8, 16.2, 18.1, 21.22, and 24.5 ns, while the *Q*_*rr*_ values were integrated to be 0.0127, 0.0536, 0.150, 0.280, and 0.405 nC, respectively. These vertical devices all exhibited a fast switching time (less than 25 ns). A considerable low reverse current is also observed in the results, which could be due to the smaller amount of stored charge in the SBDs [13]. Meanwhile, it is also clearly seen that the value of *T*_*rr*_ and *Q*_*rr*_ both increase together with the enlarging of electrode diameters, and the smallest one shows the fastest *T*_*rr*_ of 15.8 ns.

**Fig. 5:**
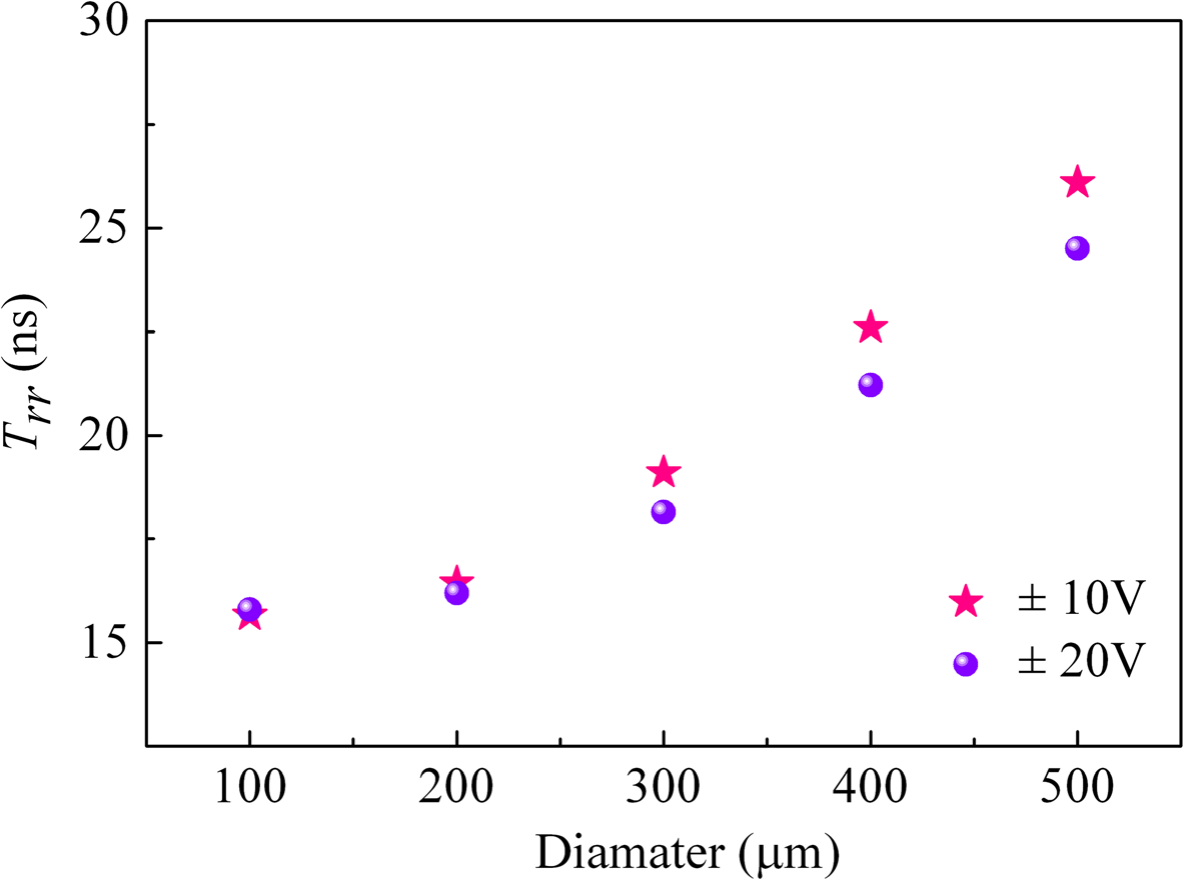
Reverse recovery time *T*_*rr*_ versus electrode diameter *d*

**Fig. 6:**
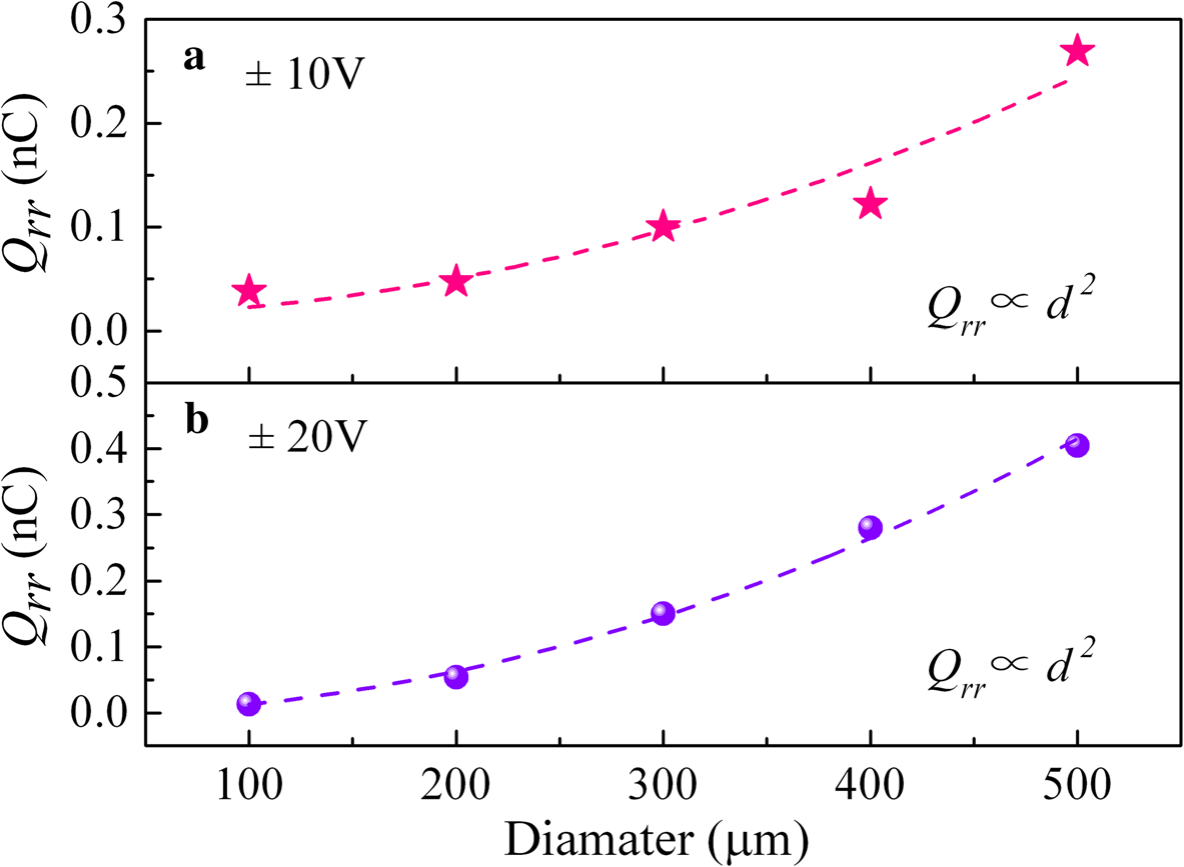
Reverse recovery charge *Q*_*rr*_ versus electrode diameter *d*

To further investigate the mechanism underlying those changes, the vertical SBDs were also measured when the voltage switched from + 10 to − 10 V. As the reverse recovery time *T*_*rr*_ versus the diode diameter *d* is plotted in Fig. 5, the value of *T*_*rr*_ for each diode was not noticeably altered. The reverse recovery charge *Q*_*rr*_ versus the *d* is displayed in Fig. 6 simultaneously, where the data of two curves point toward the same trend. Meanwhile, it is notable that the *Q*_*rr*_ of both tests show a significant positive correlation with the *d*^*2*^, that is, the electrode area.

In fact, it is reported that the reverse recovery effect should be mainly from the parasitic inductance and interface trapping of SBDs [25, 26]. Considering that the contribution of parasitic inductance is characterized in the form of oscillation current which is not obviously observed in these reverse recovery curves, thus, the changing of reverse recovery time and reverse recovery charge should have resulted from the traps [27, 28]. Since the concentration of traps is uniform in vertical SBDs, the *Q*_*rr*_ would depend on the contact area of the device and finally increase with the electrode area as shown in Fig. 6. Thus, the traps act as an electric field stopper in the interface. During the *t*_*a*_ period, the delay was strongly influenced by carrier trapping in the Schottky junction, while in the *t*_*b*_ period, reverse recovery speed is also slowed by the time for sweeping the stored charge out of the junction. These results are consistent with our previous report [29], which suggested the *RC* time constant increases with the increase of device diameter and shows a good dependency with the reverse recovery time. And a further improvement of reverse recovery characteristics could be expected from a smaller electrode or thinner drift layer in these devices.

**Fig. 7:**
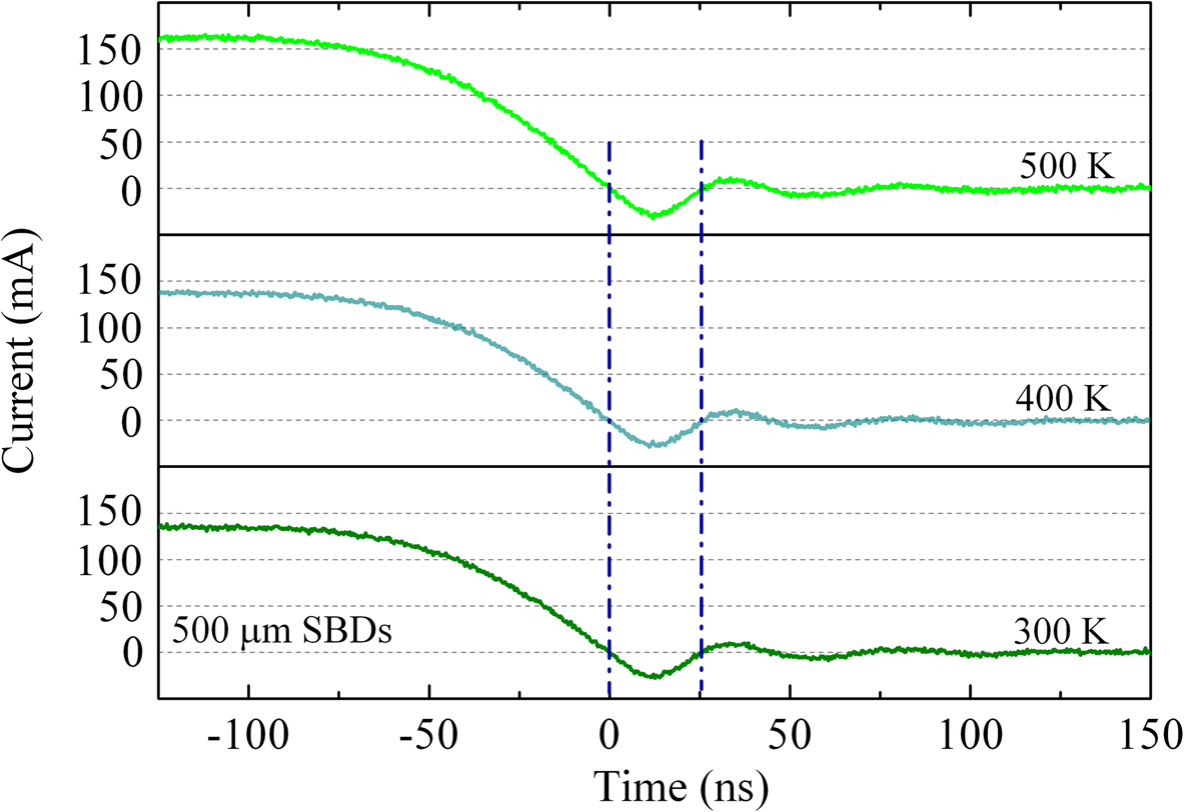
Reverse recovery characteristics for 500-μm-diameter SBDs measured at 300, 400, and 500 K, respectively

Moreover, the recovery performance of vertical SBDs is further investigated in a higher temperature. Figure 7 shows reverse recovery current for 500-μm-diameter GaN SBDs which were measured at 300, 400, and 500 K, respectively. Neither the reverse recovery time nor reverse recovery charge is observed changed with the temperature rising. These results are consistent with the above analysis, as the concentration of trap is not very sensitive to the temperature. Conversely, it is reported that the reverse recovery time of Si-based SBDs would increase by 191% as the temperature rises from 300 to 425 K [17]. Here, with a short carrier lifetime and wider bandgap, GaN SBDs are shown to provide substantial improvements in current-handling capability, reverse recovery, and energy loss. As the thermal stability of GaN-based SBDs is superior than that of traditional narrow bandgap semiconductors [30], it can be concluded that GaN is also a suitable material for switching devices applied to a high-temperature environment.

## Conclusions

In summary, we fabricated vertical GaN SBDs on Ge-doped FS GaN substrates grown by HVPE. With the material characterization and current-voltage measurements performed, it indicates that an excellent crystal quality and electronic property was obtained for the vertical SBDs. The reverse recovery characteristics were systematically investigated. The reverse recovery time was obtained to be 15.8, 16.2, 18.1, 21.22, and 24.5 ns for the 100-, 200-, 300-, 400-, and 500-μm-diameter diodes, respectively. Meanwhile, the reverse recovery time and reverse recovery charge both show a significant positive correlation with the electrode area. Our results may serve as a reference for further improving the recovery performance of GaN-based SBDs.

## Abbreviations

CL: Cathodoluminescence
*C-V*: Capacitance-voltage
DUT: Device under test
FS: Free-standing
GaN: Gallium nitride
HVPE: Hydride vapor phase epitaxy
*I-V*: Current-voltage
SBDs: Schottky barrier diodes
SEM: Scanning electron microscope
